# Enzymatic Cocktail Formulation for Xylan Hydrolysis into Xylose and Xylooligosaccharides

**DOI:** 10.3390/molecules28020624

**Published:** 2023-01-07

**Authors:** Danilo Bueno, Caroline de Freitas, Michel Brienzo

**Affiliations:** Institute for Research in Bioenergy (IPBEN), São Paulo State University (UNESP), Rio Claro 13500-230, SP, Brazil

**Keywords:** biorefinery, high-added-value products, enzymatic hydrolysis, biomass conversion, bioactive compounds

## Abstract

In the context of a biorefinery, lignocellulosic materials represent an important source of raw material for the bioconversion of cellulose, hemicellulose, and lignin into value-added products, such as xylose for fermentation, oligosaccharides, and bioplastics for packaging. Among the most abundant lignocellulosic materials in Brazil, sugarcane bagasse biomass stands out, as it is rich in cellulose and hemicellulose. In this context, through an experimental design, this study developed a robust enzyme cocktail containing xylanases and accessory enzymes to complete the hydrolysis of xylan from sugarcane bagasse, obtaining a low xylose yield and concentration (9% and 1.8 g/L, respectively, observed in experiment number 16 from the complete hydrolysis of a xylan assay), a fermentable sugar that is important in the production of second-generation ethanol, and a high xylooligosaccharides (XOS) yield and concentration (93.1% and 19.6 g/L, respectively, obtained from a xylooligosaccharides production assay); in general, xylan has prebiotic activities that favor an improvement in intestinal functions, with immunological and antimicrobial actions and other benefits to human health. In addition to completely hydrolyzing the sugarcane bagasse xylan, this enzymatic cocktail has great potential to be applied in other sources of lignocellulosic biomass for the conversion of xylan into xylose and XOS due to its enzymes content, involving both main chain and pendant groups hydrolysis of hemicelluloses.

## 1. Introduction

Hemicellulose represents a raw material in biotechnological processes, since a large surplus of lignocellulosic industrial residues from agricultural products is generated annually. These residues represent a source for bioconversion into many compounds of industrial interest, such as antioxidant compounds and oligosaccharides with bioactive potential [1]. Xylooligosaccharides can be obtained through the bioconversion of hemicellulose from different lignocellulosic biomasses, such as sugarcane bagasse [2], corn husk [3], banana pseudostem [4], among others.

Xylooligosaccharides (XOS) present several beneficial effects on human health, such as preventing caries, reducing serum cholesterol levels, and stimulating the growth of bifidobacteria in the gastrointestinal tract. Their beneficial health effects are related to their physicochemical properties, as they are moderately sweet, are stable over a wide range of pHs and temperatures, and confer organoleptic characteristics to foods. Among the benefits of oligosaccharides for human health are a reduction in cholesterol levels, maintenance of gastrointestinal health, increased calcium bioavailability, reduced risk of colon cancer, cytotoxic effects on human leukemia cells, and a beneficial effect on type 2 diabetes mellitus [5].

XOS are oligomers formed by xylose units joined by β-1,4 glycosidic bonds and are classified according to the number of repeats of xylose molecules. They are considered non-digestible oligosaccharides, and, when present in the intestine, they promote the proliferation of beneficial microorganisms for the intestinal microbiota; for this reason, they are considered prebiotics. XOS can be obtained by the hydrolysis of hemicellulose via chemical reagents, temperature, or biological agents, such as enzymatic hydrolysis [6]. These carbohydrates participate in the constitution of dietary fiber and are partially digested by humans, in which the non-digestible portions serve as food for bacteria that are part of the natural flora such as *Bifidobacteria* sp. and *Lactobacillus* sp. [7], acting in the following ways: selectively stimulating the growth and activity of one or more beneficial intestinal bacteria, improving the health of the host [8], and preventing their adhesion to gastrointestinal epithelial cells [9,10].

Oligosaccharides can be obtained through the bioconversion of different lignocellulosic biomasses, such as sugarcane bagasse [2], corn husk [3], and banana pseudostem [4], among others. Different approaches can be used for the conversion of lignocellulosic material into XOS; however, enzymatic hydrolysis stands out for its effectiveness, in addition to being used concomitantly with other techniques [11]. In the process of obtaining xylooligosaccharides via enzymatic hydrolysis, the importance of xylanases stands out, as they hydrolyze the main chain of xylan into smaller fragments containing from two to six xylose residues, which are named xylobiose, xylotriose, xylotetraose, xylopentose, and xylohexose, respectively. The fungus *Aspergillus versicolor* [12] is a wild-type microorganism that produces xylanase with a molecular mass of 19 kDa, containing 71% carbohydrates and p*I* equivalent to 5.4; when purified via ion exchange, its subsequent gel filtration shows a specific activity of 1440 U/mg and Km of 6.5 mg/mL. Xylanase from *A. versicolor* has been used in the literature to obtain XOS from different biomasses [4,13].

There are literature reports of studies that seek to obtain the deconstruction of xylan by combining the synergistic action of xylanases and accessory enzymes [14,15]. However, in these studies, the xylanolytic cocktails were not robust enough to completely hydrolyze the xylan of one or more biomasses, considering that hemicelluloses are heteropolysaccharides. The option of producing xylose or XOS is of great interest, since both products are for industrial/commercial use. In this context, this study aimed to develop an enzymatic cocktail composed of hemicellulases, both xylanases (endo-1,4-β-xylanase and β-xylosidase), which cleave the xylan main chain, and accessory enzymes (α-glucuronidase, α-L-arabinofuranosidase, acetyl xylan-esterase, and feruloyl-esterase), which hydrolyze the pendant groups of xylan. The xylanase and β-xylosidase enzymes were produced and purified from *Aspergillus versicolor*, while the accessory enzymes were purchased commercially. Subsequently, the enzymatic cocktails were evaluated in two main approaches: (i) complete hydrolysis of sugarcane bagasse xylan producing xylose and XOS; (ii) hydrolysis of xylan for the production of XOS.

## 2. Results and Discussion

### 2.1. Hemicellulose Extraction and Determination of Residual Lignin Content

A total of 200 g of xylan were extracted through an alkaline medium pretreatment under mechanical agitation, resulting in an extraction yield of about 50%, taking into account that 10 L of extraction solution were prepared and 500 g of bagasse were initially applied. The experiments on a small scale were carried out using 10 g of biomass in a reaction volume of 200 mL. After chemical characterization, the results indicated that an average of 5% corresponds to the residual lignin. The content of the residual lignin observed with this approach was higher than the 5% of the residual lignin resulting from the chemical characterization performed right after the alkaline pretreatment, following methodology published elsewhere [16], where a good yield of xylan extraction from sugarcane in alkaline pretreatment (86%) with low residual lignin content (5.9%) was reported.

### 2.2. Xylanase and β-xylosidase from A. versicolor

The xylanase from *A. versicolor* showed an enzymatic activity of 1000 IU/mL and a protein concentration equivalent to 1.98 mg/mL. Compared to data in the literature, *Aspergillus ficuum* AF-98 xylanase shows an activity of 485 IU/mL after purification with gel filtration using beechwood xylan as a substrate (FENGXIA et al., 2008). As in this study, the *Aspergillus niger* ANL 301 xylanase evaluated by Okafor et al. (2007) was produced using wheat bran as a carbon source in the culture medium. However, a low xylanase activity for *Aspergillus niger* ANL 301 was observed, equivalent to 6.47 IU/mL, different from what was obtained in this study (1000 IU/mL). Additionally, *Aspergillus niger* ANL 301 xylanase showed a protein concentration of 1.14 mg/mL, a little lower when compared to the xylanase concentration observed in this study (1.98 mg/mL).

A third study investigated the xylanase activity of *Aspergillus foetidus* in the presence of beechwood xylan as a substrate for enzymatic reaction (1%), observing a xylanase activity corresponding to 210 IU/mL (SHA and MADAMWAR, 2005). Here, using sugarcane bagasse xylan (1%) extracted via alkaline pretreatment, an *A. versicolor* xylanase activity equivalent to 1000 IU/mL was determined, which again was much higher than that observed in the literature.

The β-xylosidase that was produced and purified from *A. versicolor* showed an enzyme activity equivalent to 4.8 IU/mL in nitrophenyl-β-D-xylopyranoside substrate and a protein concentration of 0.55 mg/mL. As for the purification of β-xylosidase from *A. versicolor*, there are no data for enzyme activity published elsewhere [17].

In the literature, there are reports of specific activity equivalent to 51.30 (IU/mg protein) for β-xylosidase from *A. versicolor* when cultivated with 1% xylan as a carbon source. For the microorganism *Aspergillus nidulans* in Nitrophenyl-β-D-xylopyranoside substrate, the total enzymatic activity (IU/mL) of the β-xylosidase observed was equivalent to 44.2, 22.3, 20, 0, and 12 in different purification steps (crude extract, Q sepharose, Mono-Q and Superdex-200, respectively), which are higher values than the results reported in this study [18].

### 2.3. Complete Hydrolysis of Xylan

In the present study, a combination of enzymes was applied aiming to maximize the xylan hydrolysis. The highest yield and highest concentration of xylose observed were 9% and 1.8 g/L, with concomitant production of 90.1% and 18.22 g/L of xylooligosaccharides (Table 1). The results of the experimental design indicate that both xylose yield and concentration were low, though they were high for the xylooligosaccharides (xylobiose, xylotriose, xylotetraose, xylopentose, and xylohexose) (93.1% and 19.64 g/L) in the observed study region.

**Figure 1:**
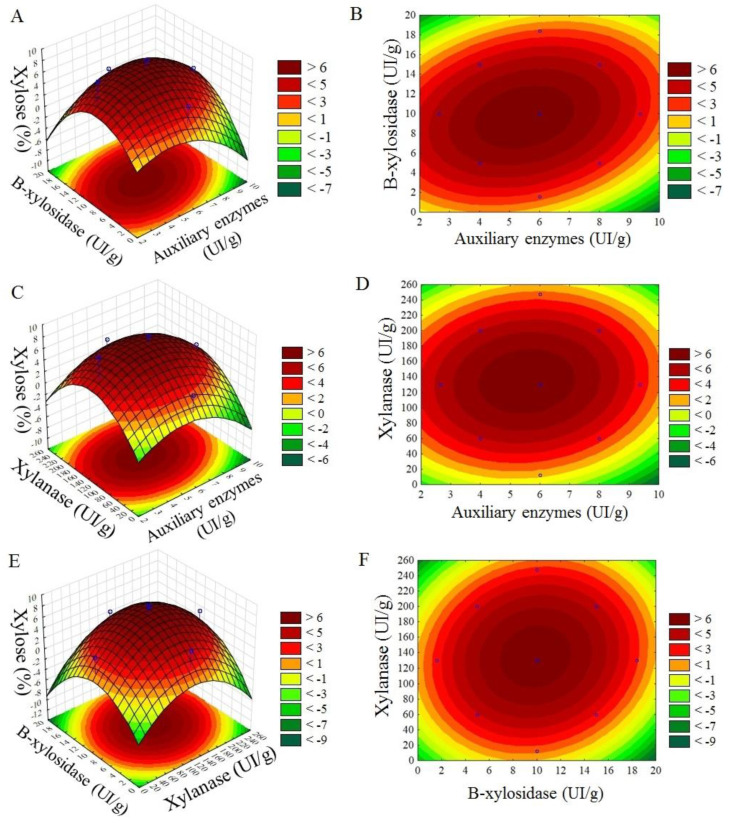
Response surface of xylose yield (%) with xylanase (IU/g), β-xylosidase (IU/g), and auxiliary enzymes (IU/g) variables. (**A**,**B**) β-xylosidase (IU/g) and auxiliary enzymes (IU/g) variables; (**C**,**D**) xylanase (IU/g) and auxiliary enzymes (IU/g) variables; (**E**,**F**) xylanase (IU/g) and β-xylosidase (IU/g) variables. For all the surfaces, the fixed variable was employed at the intermediate level (central point level).

The results of the response surface analysis for both xylose yield (%) and concentration (g/L) indicate that the best results were obtained for 130 IU/g of xylanase and 6 IU/g loads of auxiliary enzymes (Figure 1a,b). When the variables, β-xylosidase (IU/g) and the accessory enzymes (IU/g), were evaluated, the best results were observed with 10 IU/g of β-xylosidase and 6 IU/g of auxiliary enzymes (Figure 1c,d). The variables xylanase (IU/g) and β-xylosidase (IU/g) produced a higher xylose yield using 130 IU/g and 10 IU/g of enzyme loads, respectively (Figure 1e,f).

A high yield and concentration of xylooligosaccharides (93.1% and 19.6 g/L, respectively) were also observed in the experimental design of the complete xylan hydrolysis (Table 1). It is important to highlight that even in the presence of β-xylosidase, this cocktail presented better results in obtaining xylooligosaccharides than the experimental design itself, which used an enzyme cocktail with the absence of β-xylosidase, since the production of xylose was not the target of the analysis. Hypothetically, a higher β-xylosidase load could lead to higher xylose production (in yield and concentration), thus reducing XOS production (yield and concentration). On the other hand, the xylose was not increased, and probably a higher amount of β-xylosidase could be necessary.

In the experimental design to obtain the complete hydrolysis of xylan, the highest yield and concentration of xylooligosaccharides observed were 93.1% and 19.6 g/L, respectively, with concomitant production of 4% and 0.8 g/L of xylose. According to the response surface, the highest value for XOS yield (>90%) was observed when 130 IU/g of xylanase, 20 IU/g of β-xylosidase, and 7 IU/g of accessory enzymes were used.

**Figure 2:**
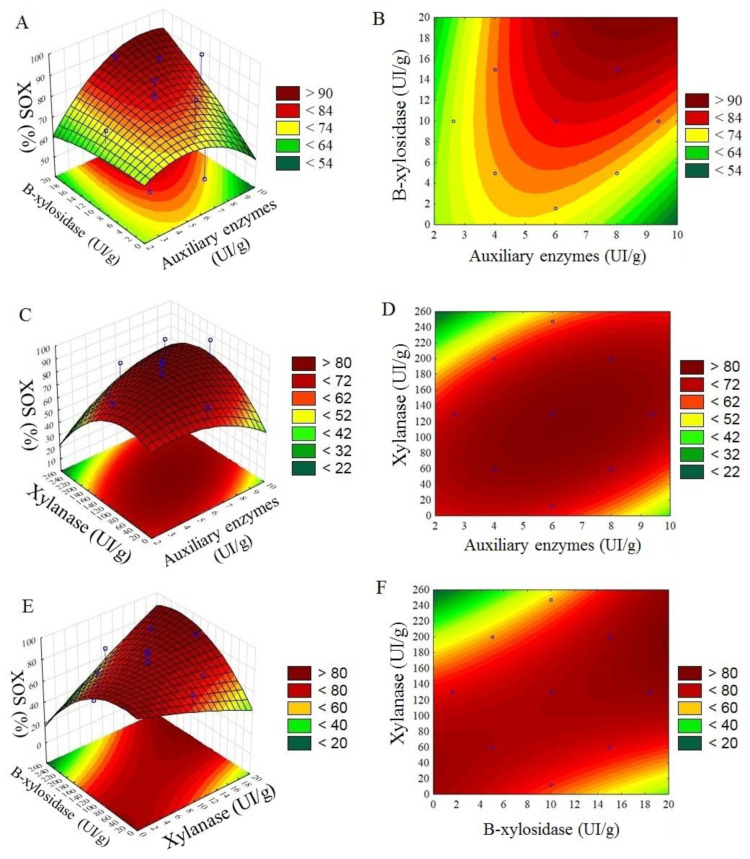
Response surface of xylooligosaccharides yield (%) to experimental design to obtain complete hydrolysis of xylan using xylanase (IU/g), β-xylosidase (IU/g), and auxiliary enzymes (IU/g) variables. (**A**,**B**) β-xylosidase (IU/g) and auxiliary enzymes (IU/g) variables; (**C**,**D**) xylanase (IU/g) and auxiliary enzymes (IU/g) variables; (**E**,**F**) xylanase (IU/g) and β-xylosidase (IU/g) variables.

Through the results, it was also possible to observe that the maximum xylanase load (247.7 IU/g) generated the highest yield and XOS concentration results (93.1% and 19.6 g/L, respectively), while the lower xylanase load (12.2 IU/g) generated low yield and XOS concentration results (76.6% and 15.3 g/L). Additionally, the maximum load of β-xylosidase (15 IU/g) generated XOS yields ranging from 40% to 74.5% and concentrations from 12 to 17.7 g/L, while the lower β-xylosidase load (1.5 IU/g) generated a high yield of XOS (93%) and also a high concentration (19 g/L). The intermediate auxiliary enzymes load (central point) generated a higher XOS yield in combination with xylanase (Figure 2). Probably there was a positive interaction between these enzymes, resulting in better XOS production.

In relation to the results obtained for xylose, a study in the literature showed that 22.5% xylose was obtained when sugarcane bagasse was submitted to an alkaline pretreatment and subsequent enzymatic hydrolysis for 24 h using 5 IU/g of xylanase [19]. In the present study, to obtain 9% of xylose (better result), 130 IU/g of xylanase were used, but other enzymes were added, namely β-xylosidase (10 IU/g) and accessory enzymes (6 IU/g), in a 24 h reaction with xylan as a substrate that was also submitted to alkaline pretreatment. However, despite the low xylose yield observed, high xylooligosaccharides yields were obtained in the same experiment, as described above.

A second study produced 4.26% of xylose, also with sugarcane bagasse biomass submitted to alkaline pretreatment and subsequently hydrolyzed using 500 U/g of xylanase for periods longer than 24 h of reaction in a bioreactor [20]. For comparison purposes, this present study produced more than double this xylose yield (9%), using a much lower enzyme load (130 IU/g) for a shorter reaction time (24 h).

### 2.4. Xylooligosaccharides Production

**Figure 3:**
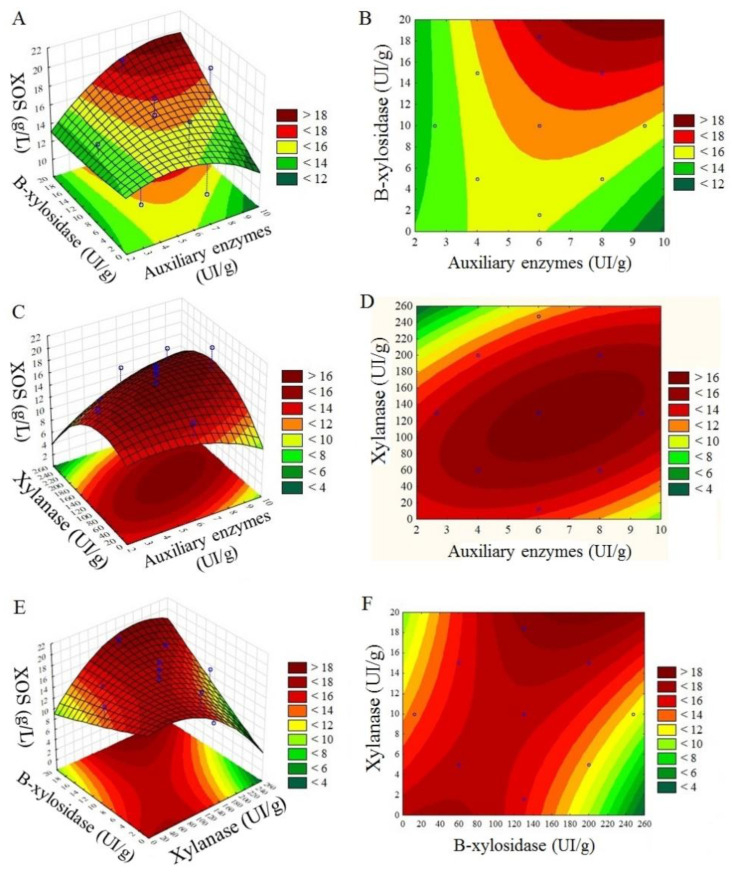
Response surface of xylooligosaccharides yield (%) and concentration (g/L) with xylanase (IU/g) and auxiliary enzymes (IU/g) variables. (**A**,**B**) Xylooligosaccharides yield (%) with xylanase (IU/g) and auxiliary enzymes (IU/g) variables; (**C**,**D**) xylooligosaccharides concentration (g/L) with xylanase (IU/g) and auxiliary enzymes (IU/g) variables; (**E**,**F**) xylooligosaccharides concentration (g/L) with xylanase (IU/g) and *β*-xylosidase (IU/g).

The best results observed for both the yield (around 84%) and concentration (16.8 g/L) of xylooligosaccharides occurred when low enzymatic loads of auxiliary enzymes (2 IU/g) were used, with xylanase loads ranging from 20 IU/g to 80 IU/g, respectively (Table 2). The results of response surface analysis for both xylooligosaccharide yield (%) (Figure 3) and concentration (g/L) indicate that the ideal xylanase enzymatic load corresponds to 100 (IU/g), which is higher than the study region observed in this study (7.47 to 92.43 IU/g), while low loads of auxiliary enzymes would be ideal to obtain better results.

The maximum xylanase load (92.4 IU/g) generated a yield of 81.2% XOS and a concentration of 16.2 g/L, while the lowest xylanase load (7.5 IU/g) generated a yield and an XOS concentration equivalent to 80.3% and 16 g/L, respectively. For accessory enzymes, the maximum load (11.6 IU/g) generated a yield of 68.3% and a concentration of 13.6 g/L of XOS, while the lower load (0.3 IU/g) presented a yield and concentration of XOS equivalent to 80.6% and 16.1 g/L of XOS.

A study also used *A. versicolor* xylanase [21], the same used in this study, to obtain xylooligosaccharides from banana pseudostem biomass. Various xylanase enzyme loads were used (10, 30, 60, 100, and 200 IU/g), and 52.27%, 60.98%, 58.0%, 57.14%, and 62.91% of xylooligosaccharide yield (xylobiose, xylotriose, xylotetraose, xylopentose, and xylohexose, respectively) were obtained, with concentrations (g/L) equivalent to 9.44, 11.02, 10.48, 10.32, and 11.36, respectively. In this present study, all enzyme loads evaluated for xylanase (IU/g) led to yields ranging from 55.4% to 84% and concentrations ranging from 11.1 to 16.8 g/L, considering that this study used a complete enzyme cocktail with xylanase and also auxiliary enzymes. Regarding the polymerization degree for the xylooligosaccharides produced, in this study, yields equivalent to 9.9% (xylobiose), 39.6% (xylotriose), 21.2% (xylotetraose), and 30.2% (xylopentose and xylohexose) were observed, yields that were mostly higher compared to those observed in another study [4], where yields of 0.55% (xylobiose), 0.53% (xylotriose), 3.75% (xylotetraose), and 6.97% (xylopentose and xylotetraose) were reported.

A second study [13] used the same xylanase and *A. versicolor* [21] as this present study for the production of xylooligosaccharides from sugarcane bagasse and leaf biomass. Enzymatic loads of xylanase ranging from 15.5 to 100 UI/g were used, obtaining a maximum XOS yield of 67.43% for bagasse and 69.71% for sugarcane leaf. The maximum XOS concentration was 19.91 g/L for sugarcane bagasse and 21.48 g/L for sugarcane leaf. The polymerization degree of xylooligosaccharides described was 3.74% for xylobiose and 64.26% for xylotriose, xylotetraose, xylopentose, and xylohexose together, also mostly lower than those observed in this study.

**Figure 4:**
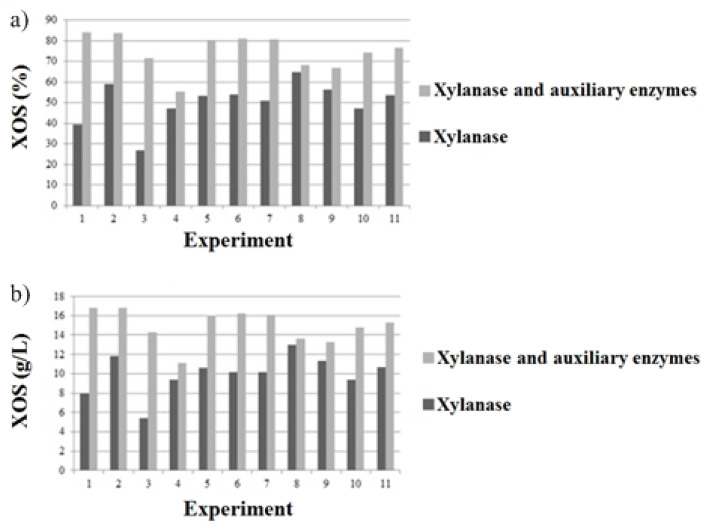
Xylooligosaccharides production by xylanase and auxiliary enzymes approach (IU/g) and only xylanase approach (IU/g). (**a**) Xylooligosaccharides yield (%) and (**b**) xylooligossaccharides concentration (g/L). The *x*-axis represents the number of experiments referred to in Table 2 compared to the absence of auxiliary enzymes.

With *Bacillus subtilis* (using xylanase and not purified *β*-xylosidase), a XOS yield of 3.25% was observed in a 72 h growing period with wheat middlings as the substrate [22]. Moreover, polymerization degres of 0.37% for xylobiose, 1.78% for xylotriose, 1.47% for xylotetraose, and 0.9% for xylopentose and xylohexose were observed, which are also lower than those observed in the present study. Here, it is noteworthy that the use of the auxiliary enzymes α-L-arabinofuranosidase, α-glucuronidase, acetyl xylan-esterase, and feruloyl esterase was essential to obtain better results in the production of xylooligosaccharides. The same xylanase loads (IU/g) were evaluated in the absence and presence of auxiliary enzymes (Figure 4), and the results clearly indicate that there is an increase in the yield (%) and concentration (g/L) of XOS when accessory enzymes are used together with xylanase compared to xylanase alone.

The biomass is recalcitrant and required a series of strategies to convert it into value-added molecules [23]. A biorefinery approach appears as a solution for better use of biomass components, resulting in a different process such as xylose for furfural [24] or fermentation, XOS, and even the xylan solubilization residue being used for briquettes (energy densification) [25]. Xylose and XOS can be produced from different types of waste biomass [26], by applying an enzymatic or acid approach [27,28]. However, the present study showed the advantage of using combined hemicelluloses hydrolysis enzymes for XOS production.

## 3. Methodology

### 3.1. Hemicellulose Extraction

Hemicellulose extraction was carried out with sugarcane bagasse treated with 0.2% (*m*/*v*) ethylenediamine tetraacetic acid (EDTA) solution for 1 h at 90 °C to remove metals. Extraction was performed with conditions optimized for bagasse, 6% H_2_O_2_ (*m*/*v*) at 25 °C for 4 h [16]. Ten grams of bagasse were placed in 1 L flasks, followed by the addition of the prepared reagents in a volume of 200 mL, the pH was adjusted to 11.6 with 5 mol/L NaOH, and the medium was stirred at 80 rpm. After the reaction, the material was filtered through filter paper. The pH of the filtrate was immediately corrected to 6 with the addition of 6 mol/L HCl. In the xylan solution, 3 vol ethanol were added. After xylan precipitation, the liquid fraction (75% ethanol) was changed 3 times for washing. The xylan was separated from the liquid fraction and oven-dried at 45 °C.

### 3.2. Evaluation of Lignin Residual Content

The methodology for bagasse, which was applied to hemicellulose [16], consisted of hydrolyzing approximately 300 mg of biomass and adding 1.5 mL of 72% H_2_SO_4_ (*m*/*m*); the reaction occurred at 45 °C for 7 min. The reaction was stopped with the addition of 45 mL of distilled water. This mixture was autoclaved at 121 °C for 30 min [29]. The contents were filtered through a porous plate filter (number 4), previously tared. The solid residue was washed with distilled water and dried in an oven at 105 °C until constant weight for the determination of insoluble lignin (Klason).

### 3.3. Purification of Xylanase and β-xylosidase from A. versicolor

The fungus *A. versicolor* was initially cultivated for 7 days at 30 °C in petro plates containing a solid medium from Vogel (1965). Then, 1.0 mL of the spore suspension (5 × 10^7^ spores/mL) were inoculated for 5 days in a VOGEL (1965) liquid medium containing 1% wheat bran, following the established methodology [17]. The material was filtered on Whatman No. 1 paper and was dialyzed against 50 mmol/L Tris-HCl buffer to remove impurities. Then, the crude extract was subjected to ion exchange purification [29]. Thus, 15 mL of SEPHADEX A-50 resin (GE HEALTHCARE) and 150 mL of the enzymatic extract were used in 250 mL Erlenmeyer submitted to 10 °C under light agitation for a period of 24 h in a refrigerated incubator (MARCONI MA 830/A). After a period of 24 h, the mixture was filtered through filter paper (7 cm in diameter, pores of 14 μm) with the aid of a vacuum pump (MARCONI MA057). The gel filtration was performed using SEPHADEX G-75 resin (GE HEALTHCARE), completing the purification process.

### 3.4. Determination of Enzymatic Activity and Protein Quantification

The xylanase activity of *A. versicolor* was determined following the established method [30], in a reaction at 50 °C for 5 min, using 1% xylan as substrate. The β-xylosidase activity of *A. versicolor* was determined following an established methodology [18], in a reaction at 50 °C for 5 min using Nitrophenyl-β-D-xylopyranoside (Megazyme) as substrate. The auxiliary enzymes used in this study were purchased from the company Megazyme, and their information is gathered in Table 3.

### 3.5. Complete Xylan Hydrolysis and Xylooligosaccharides Production

Enzyme cocktails were evaluated to determine the enzyme load (IU/g of xylan/substrate) required for the complete hydrolysis of hemicellulose. An experimental design was carried out by varying the enzymatic loads of xylanase, β-xylosidase, and auxiliary enzymes (α-glucuronidase, α-L-arabinofuranosidase, acetyl xylan esterase, and feruloyl esterase) in order to obtain complete hydrolysis of xylan (Table 4).

A second experimental design was carried out to obtain xylooligosaccharides, excluding the use of the β-xylosidase enzyme (Table 5). Parameters such as reaction time (24 h), temperature (50 °C), stirring (100 rpm), and reaction volume (1 mL) were fixed for all reactions in both approaches. Samples were filtered through 0.22 μm syringe filters and evaluated by HPLC using the Aminex HPX-87C BIO-RAD column (300 × 7.8 mm) [21]. Finally, another assay was performed, applied only to xylanase enzymes with the same enzyme loads (IU/g) used for XOS production (Table 5), for comparison with the approach where xylanase and auxiliary enzymes were used together.

### 3.6. Statistical Analyses

Statistical analysis of the effects of each variable to obtain complete hydrolysis of xylan and the production of xylooligosaccharides was performed using Statistica 8 software, considering the confidence level of 95% (*p* > 0.5), with ANOVA statistical analysis, and the respective response surface graphics for both complete hydrolyses of xylan and production of xylooligosaccharides assays were generated.

The results of the second-order ANOVA statistical analysis for the experimental design of complete hydrolysis of xylan indicated that it would be appropriate to perform a first-order analysis (Table S1), while ANOVA statistical analysis was more appropriate for the results of the experimental design for obtaining second-order XOS (Table S2).

## 4. Conclusions

This study is a pioneer in using a robust enzyme cocktail containing both the enzymes that cleave the hemicellulose main chain and most of the existing auxiliary enzymes that hydrolyze the pendant groups of hemicellulose, such as arabinose, glucuronic acid, acetic acid, and ferulic acid. A complete hydrolysis of xylan from sugarcane bagasse is carried out and still produces xylooligosaccharides, one of the xylan derivatives with value-added. High yields (93.1%) and concentrations (19.6 g/L) of xylooligosaccharides (xylobiose, xylotriose, xylotetraose, xylopentose, and xylohexose) were observed in this study. It is important to emphasize that this enzyme cocktail was developed by taking into account that hemicellulose is a heteropolysaccharide; that is, its content may vary from biomass to biomass. Therefore, in addition to being used in the bioconversion of xylan from sugarcane bagasse into xylose and xylooligosaccharides, this enzyme cocktail may also be used for the bioconversion of hemicellulose from other sources of lignocellulosic raw material.

## Figures and Tables

**Table 1:** Experimental design to obtain the complete hydrolysis of xylan evaluating xylanse, β-xylosidase, and accessory enzymes.

Assay	*Xylanase* (IU/g)	*β-xylosidase* (IU/g)	Accessory Enzymes (IU/g) *	X2 (%)	X3 (%)	X4 (%)	X5 + X6 (%)	XOS (%)	XOS (g/L)	Xylose (%)	Xylose (g/L)	Total Sugars (%)	Total Sugars (g/L)
1	60	5	4	9.9	28.1	13.4	19.0	70.4	14.1	1.5	0.3	71.9	14.3
2	200	5	4	5.0	22.2	11.6	11.1	49.9	10.0	1.4	0.1	51.3	10.0
3	60	15	4	8.0	29.0	15.5	22.0	74.5	14.9	0.2	0.0	74.7	14.9
4	200	15	4	3.0	30.1	12.2	17.5	62.8	12.5	1.1	0.2	63.9	12.8
5	60	5	8	14.0	-	21.5	30.7	66.2	13.2	0.2	0.1	66.4	13.3
6	200	5	8	1.4	-	17.2	24.5	43.1	8.6	0.5	0.1	43.6	8.7
7	60	15	8	14.2	-	18.8	26.8	59.8	12.0	1.0	0.2	60.8	12.2
8	200	15	8	39.5	-	20.2	28.8	40.0	17.7	3.6	0.7	43.6	18.4
9	12.2	10	6	31.6	-	18.5	26.5	76.6	15.3	3.7	0.7	80.3	16.0
10	247.7	10	6	25.6	33.5	14.0	20.0	93.1	19.6	4.0	0.8	97.1	19.7
11	130	1.5	6	22.1	35.8	15.2	21.7	93.0	19.0	5.4	1.0	98.4	20.0
12	130	18.41	6	24.2	35.2	12.6	18.0	90.0	19.3	3.3	0.6	93.3	20.0
13	130	10	2.6	14.0	28.8	14.9	21.2	78.9	15.7	8.9	1.7	87.8	17.5
14	130	10	9.3	30.0	-	16.9	24.0	70.9	14.1	4.1	0.8	75.0	15.0
15	130	10	6	15.0	33.2	15.9	22.6	85.0	17.3	8.0	1.6	94.3	18.9
16	130	10	6	16.0	33.3	16.8	24.0	90.1	18.2	9.0	1.8	99.1	20.0
17	130	10	6	14.0	32.2	15.0	21.3	83.0	16.5	7.1	1.4	89.6	17.9

* *α-L-arabinofuranosidasse*, *α-glucuronidase, acetil xylan-esterase*, and *feruloil esterase*. X2: xylobiose; X3: xylotriosis; X4: xylotetratose; X5 + X6: xylopentose and xylohexose.

**Table 2:** Xylooligosaccharides production obtained through experimental design evaluating xylanase and accessory enzymes.

Experiment	*Xylanase* (IU/g)	Accessory Enzymes (IU/g) *	X2	X3	X4	X5 + X6	Total XOS (%)	Total XOS (g/L)
1	20	2	9.9	23.1	21.0	30.0	84.0	16.8
2	80	2	5.5	39.6	16.0	22.8	83.9	16.8
3	20	10	8.8	19.6	17.8	25.4	71.6	14.3
4	80	10	5.5	18.6	16.0	15.3	55.4	11.1
5	7.5	6	5.6	23.3	21.2	30.2	80.3	16.0
6	92.4	6	7.0	23.2	21.0	30.0	81.2	16.2
7	50	0.3	3.1	33.1	18.3	26.1	80.6	16.1
8	50	11.6	6.8	19.2	17.4	24.9	68.3	13.6
9	50	6	6.1	18.9	17.1	24.5	66.6	13.3
10	50	6	3.8	42.6	14.2	13.6	74.2	14.8
11	50	6	3.1	39.4	17.4	16.7	76.6	15.3

* *α-L-arabinofuranosidasse*, *α-glucuronidase, acetil xylan-esterase*, and *feruloil esterase*. X2: xylobiose; X3: xylotriosis; X4: xylotetratose; X5 + X6: xylopentose and xylohexose.

**Table 3:** Commercial auxiliary enzymes activity and optimum temperature and pH.

Enzyme	Microorganism	Activity (IU/mL)	Specific Activity (IU/mg Protein)	Temperature (°C)	pH
acetyl xylan esterase	*Orpinomyces* sp.	1000	36	40	6.7
α-glucuronidase	*Geobacillus stearothermophilus*	100	40	70	7.0
α-L arabinofuranosidase	*Bacteroides ovatus*	1000	575	40	6.5
feruloyl esterase	Rumen microorganisms	400	30	40	6.5

**Table 4:** Central composite design (with rotatable/star points) experiments for determination of complete hydrolysis of xylan.

Experiment	*Xylanase* (IU/g)	*β-xylosidase* (IU/g)	Auxiliary Enzymes (IU/g) *
1	60	5	4
2	200	5	4
3	60	15	4
4	200	15	4
5	60	5	8
6	200	5	8
7	60	15	8
8	200	15	8
9	12.27	10	6
10	247.73	10	6
11	130	1.59	6
12	130	18.41	6
13	130	10	2.64
14	130	10	9.36
15	130	10	6
16	130	10	6
17	130	10	6

* Auxiliary enzymes were added with the same enzymatic load.

**Table 5:** Experimental design for xylooligosaccharides production.

Experiment	*Xylanase* (IU/g)	Auxiliary Enzymes (IU/g) *
1	20	2
2	80	2
3	20	10
4	80	10
5	7.57	6
6	92.43	6
7	50	0.34
8	50	11.66
9	50	6
10	50	6
11	50	6

* Auxiliary enzymes were added with the same enzymatic charge.

## Supplementary Materials

The following supporting information can be downloaded at: https://www.mdpi.com/article/10.3390/molecules28020624/s1. Table S1: ANOVA statistical analysis of XOS yield (%) from experimental design of complete hydrolysis of xylan (R2 = 0.98934). Table S2: ANOVA statistical analysis of XOS yield (%) from experimental design of xylooligosaccharides obtaining (R2 = 0.75176).

## Sample Availability

Samples of the compounds are not available from the authors.

## References

[1] Akpinar O., Erdogan K., Bakir U., Yilmaz L. (2010). Comparison of acid and enzymatic hydrolysis of tobacco stalk xylan for preparation of xylooligosaccharides. LWT Food Sci. Technol..

[2] Zhou X., Xu Y. (2019). Integrative process for sugarcane bagasse biorefinery to co-produce xylooligosaccharides and gluconic acid. Bioresour. Technol..

[3] Qian S., Zhou J., Chen X., Ji W., Zhang L., Hu W., Lu Z. (2020). Evaluation of an efficient fed-batch enzymatic hydrolysis strategy to improve production of functional xylooligosaccharides from maize straws. Ind. Crops Prod..

[4] Freitas C., Terrone C.C., Masarin F., Carmona E.C., Brienzo M. (2021). In vitro study of the effect of xylooligosaccharides obtained from banana pseudostem xylan by enzymatic hydrolysis on probiotic bacteria. Biocatal. Agric. Biotechnol..

[5] Ando H., Ohba H., Sasaki T., Takamine K., Kamino Y., Moriwaki S., Bakalova R., Uemura Y., Hatate Y. (2004). Hot-compressed-water decomposed products from bamboo manifest a selective cytotoxicity against acute lymphoblastic leukemia cells. Toxicol. In Vitro.

[6] Freitas C., Carmona E., Michel B. (2019). TOP Xylooligosaccharides production process from lignocellulosic biomass andbioactive effects. Bioact. Carbohydr. Diet. Fibre.

[7] Giese E.C., Hirosi T., Silva M.D.L.C., Silva R., Barbosa A.M. (2011). Production, properties and applications of oligosaccharides. Semin. Ciênc. Agrárias.

[8] Singh R.P., Benerjee J., Arora A. (2015). Prebiotic potential of oligosaccharides: A focus on xylan derived oligosaccharides. Bioact. Carbohydr. Dietery Fibre.

[9] Holzapfel W.H., Schillinger U. (2002). Introduction to pre-and probiotics. Food Res. Int..

[10] Shimizu M., Hachimura S. (2011). Gut as a target for functional food. Trends Food Sci. Technol..

[11] He M.X., Wang J.L., Qin H., Shui Z.X., Zhu Q.L., Wu B., Tan F., Pan K., Hu Q., Dai L. (2014). Bamboo: A new source of carbohydrate for biorefinery. Carbohydr. Polym..

[12] Carmona E., Rochetto-Braga M.R., Pizzirani-Kleiner A.A., Jorge J.A. (1998). Purification and biochemical characterization of an endoxylanase from Aspergillus versicolor. FEMS Microbiol. Lett..

[13] Forsan C.F., de Freitas C., Masarin F., Brienzo M. (2021). Xylooligosaccharide production from sugarcane bagasse and leaf using *Aspergillus versicolor* endoxylanase and diluted acid. Biomass Convers. Biorefinery.

[14] Yi Z., Su X., Asangba A.E., Abdel-Hamid A.M., Chakraborty S., Dodd D., Stroot P.G., Mackie R.I., Cann I. (2022). Xylan Deconstruction by Thermophilic *Thermoanaerobacterium bryantii* Hemicellulases Is Stimulated by Two Oxidoreductases. Catalysts.

[15] Ávila P.F., Cairo J.P.L.F., Damásio A., Forte M.B.S., Goldbeck R. (2020). Xylooligosaccharides production from a sugarcane biomass mixture: Effects of commercial enzyme combinations on bagasse/straw hydrolysis pretreated using different strategies. Food Res. Int..

[16] Brienzo M., Siqueira A.F., Milagres A.M. (2009). Search for optimum conditions of sugarcane bagasse hemicellulose extraction. Biochem. Eng. J..

[17] Carmona E.C., Pizzirani-Kleiner A.A., RosimMonteiro R.T., Jorge J.A. (1998). Xylanase production by *Aspergillus versicolor*. J. Basic Microbiol..

[18] Kumar S., Ramón D. (1996). Purification and regulation of the synthesis of a β-xylosidase from *Aspergillus nidulans*. FEMS Microbiol. Lett..

[19] Quintero L.P., de Souza N.P.Q., Milagres A.M.F. (2020). The Effect of Xylan Removal on the High-Solid Enzymatic Hydrolysis of Sugarcane Bagasse. Bioenergy Res..

[20] Raj K., Krishnan C. (2020). Improved co-production of ethanol and xylitol from low-temperature aqueous ammonia pretreated sugarcane bagasse using two-stage high solids enzymatic hydrolysis and Candida tropicalis. Renew. Energy.

[21] Freitas C., Terrone C.C., Carmona E.C., Brienzo M. (2020). Evaluation of xylooligosaccharides effect on the growth of probiotic microorganisms. Braz. J. Dev..

[22] Reque P.M., Pinilla C.M.B., Gautério G.V., Kalil S.J., Brandelli A. (2019). Xylooligosaccharides production from wheat middlings bioprocessed with Bacillus subtilis. Food Res. Int..

[23] Hou Q., Qi X., Zhen M., Qian H., Nie Y., Bai C., Zhang S., Bai X., Ju M. (2021). Biorefinery roadmap based on catalytic production and upgrading 5-hydroxymethylfurfural. Green Chem..

[24] Nie Y., Hou Q., Li W., Bai C., Bai X., Ju M. (2019). Efficient Synthesis of Furfural from Biomass Using SnCl_4_ as Catalyst in Ionic Liquid. Molecules.

[25] Ndumbo M., de Freitas C., de Conti A.C., Brienzo M. (2022). Biomass biorefinery for biopolymers isolation, fermentable sugars, and briquettes production. Biomass Conv. Bioref..

[26] Pereira B.S., de Freitas C., Contiero J., Brienzo M. (2022). Enzymatic Production of Xylooligosaccharides from Xylan Solubilized from Food and Agroindustrial Waste. Bioenerg. Res..

[27] Forsan C.F., Schmatz A., Masarin F., Brienzo M. (2022). Xylooligosaccharide production by optimized sulfuric, acetic acid, and liquid hot water treatment of sugarcane leaves. Biomass Conv. Bioref..

[28] Freitas C., Brienzo M. (2022). Enzymatic Hydrolysis Applied to Banana Pseudostem Biomass Compared to Solubilized Xylan for Xylooligosaccharides Production with High Substrate Concentration. Bioenerg. Res..

[29] Golveia E.R., Nascimento R.T., Souto-Maior A.M., Rocha G.J.M. (2009). Validação de metodologia para a caracterização química de bagaço de cana-de-açúcar. Química Nova.

[30] Miller G.L. (1959). Use of dinitrosalicylic acid reagent for determination of reducing sugar. Anal. Chem..

