# Supplementary material for: Enzymatic Cocktail Formulation for Xylan Hydrolysis into Xylose and Xylooligosaccharides
Source: Molecules. 2023 Jan 7;28(2):624. doi: 10.3390/molecules28020624 (PMC9862374; doi:10.3390/molecules28020624)
Supplement: Supplementary file 1 [file molecules-28-00624-s001.zip › molecules-1958066-supplementary.pdf]

Supplementary table 1. ANOVA statistical analysis of XOS yield (%) from experimental design of complete hydrolysis of xylan ($R^2 = 0.98934$).

Factor	SS	Df	MS	F	p
Curvature	1653.502	1	1653.502	110.2090	0.008952
(1)Xylanase	705.001	1	705.001	46.9896	0.020625
(2)β-xylosidase	7.031	1	7.031	0.4686	0.564295
(3)Auxiliary enzymes	294.031	1	294.031	19.5977	0.047426
1 by 2	18.301	1	18.301	1.2198	0.384496
1 by 3	14.311	1	14.311	0.9539	0.431737
2 by 3	87.781	1	87.781	5.8508	0.136722
1*2*3	3.781	1	3.781	0.2520	0.665469
Error	30.007	2	15.003		
Total SS	2813.747	10			

Supplementary table 2. ANOVA statistical analysis of XOS yield (%) from experimental design of xylooligosaccharides obtaining ($R^2 = 0.75176$).

Factor	SS	Df	MS	F	p
(1)Xilanase(L)	28.3371	1	28.3371	0.74060	0.428794
Xilanase(Q)	56.5161	1	56.5161	1.47707	0.278484
(2)Auxiliary enzymes(L)	424.2537	1	424.2537	11.08807	0.020782
Auxiliary enzymes(Q)	0.0012	1	0.0012	0.00003	0.995669
1L by 2L	65.0442	1	65.0442	1.69996	0.249091
Error	191.3108	5	38.2622		
Total SS	770.6558	10			
